# MWC allosteric model explains unusual hemoglobin-oxygen binding curves from sickle cell drug binding

**DOI:** 10.1016/j.bpj.2021.04.024

**Published:** 2021-04-29

**Authors:** Eric R. Henry, Julia Harper, Kristen E. Glass, Belhu Metaferia, John M. Louis, William A. Eaton

**Affiliations:** 1 National Institutes of Health, Bethesda, Maryland

## Abstract

An oxygen-affinity-modifying drug, voxelotor, has very recently been approved by the FDA for treatment of sickle cell disease. The proposed mechanism of action is by preferential binding of the drug to the R quaternary conformation, which cannot copolymerize with the T conformation to form sickle fibers. Here, we report widely different oxygen dissociation and oxygen association curves for normal blood in the presence of voxelotor and interpret the results in terms of the allosteric model of Monod, Wyman, and Changeux with the addition of drug binding. The model does remarkably well in quantitatively explaining a complex data set with just the addition of drug binding and dissociation rates for the R and T conformations. Whereas slow dissociation of the drug from R results in time-independent dissociation curves, the changing association curves result from slow dissociation of the drug from T, as well as extremely slow binding of the drug to T. By calculating true equilibrium curves from the model parameters, we show that there would be a smaller decrease in oxygen delivery from the left shift in the dissociation curve caused by drug binding if drug binding and dissociation for both R and T were rapid. Our application of the Monod, Wyman, and Changeux model demonstrates once more its enormous power in explaining many different kinds of experimental results for hemoglobin. It should also be helpful in analyzing oxygen binding and in vivo delivery in future investigations of oxygen-affinity-modifying drugs for sickle cell disease.

## Significance

The allosteric model of Monod, Wyman, and Changeux (MWC) has been widely used to explain cooperative effects in multisubunit proteins. Studies on hemoglobin remain the research paradigm for applications of the MWC model. According to MWC, cooperative binding of oxygen to hemoglobin results from a shift in the population of the low-affinity (T) conformation to the high-affinity (R) conformation as successive molecules of oxygen bind. Voxelotor, a recently approved FDA drug for sickle cell disease, acts by preferential binding to the nonpolymerizing R conformation to reduce sickling. The resulting oxygen-binding curves with drug bound reported here are biphasic and time dependent. We explain this complex behavior quantitatively with a straightforward extension of the MWC model to include drug binding and dissociation rates, which provides a guide for future drug development for sickle cell disease using this strategy.

## Introduction

Understanding oxygen binding by hemoglobin and its relation to the pathogenesis and treatment of sickle cell disease has a long and interesting history (1, 2, 3). The first accurate measurements that showed oxygen binding to be cooperative and dependent on acidity (the Bohr effect) were made 117 years ago by the physiologist Christian Bohr, the father of the famous theoretical physicist Niels Bohr, and co-workers (4). 50 years ago, Max Perutz proposed an explanation for both cooperative binding and the Bohr effect in terms of the three-dimensional structure of hemoglobin determined by x-ray crystallography, a study that gave birth to the field of structure-function relations in biochemistry (5). Shortly thereafter, Attila Szabo and Martin Karplus developed a mathematical model (6), which showed that the Perutz mechanism explained the most important biochemical data existing at the time and is consistent with the two-state allosteric model of Jacques Monod, Jeffries Wyman, and Jean-Pierre Changeux (MWC), one of the most highly cited theoretical works in all of biology (2,7). In the MWC model, binding to both R and T conformations is noncooperative (8,9). Cooperative binding produces a sigmoid-shaped binding curve that results from the shift in the quaternary conformation population from low-affinity T to high-affinity R as successive molecules of oxygen bind. Although the measurements of Bohr et al. required many minutes, they correctly assumed that their results were of direct physiological relevance, even though oxygen binding and dissociation in vivo occur on the seconds timescale. Later kinetic studies justified their assumption by showing that these chemical reactions are subsecond (10,11). Now that increasing the affinity of hemoglobin S is being widely used as a therapeutic strategy to treat sickle cell disease (reviewed in (12)), it is important to revisit the issue of the relation between in vitro and in vivo oxygen binding and dissociation.

It has been known since the very early reports of sickle cell disease that sickling is highly dependent on oxygen pressure (13) and, more recently, that the kinetics of sickling are exquisitely sensitive to the average number of oxygen molecules bound to the hemoglobin S tetramer, usually expressed as the fractional saturation with oxygen (14, 15, 16, 17, 18, 19, 20). Consequently, increasing the oxygen affinity of hemoglobin S to reduce sickling in the microcirculation of the tissues has been a long-considered (12,21,22) but controversial strategy for treating sickle cell disease (23). The molecular rationale for this strategy is that shifting the quaternary equilibrium from the polymerizing T conformation to the R conformation (14,22,24, 25, 26, 27), which cannot copolymerize (16,17,28), will reduce sickling. The strategy is controversial because it is not clear whether oxygen delivery to the tissues will be increased sufficiently from the expected decrease in the frequency of sickling-induced vaso-occlusion to overcome the decrease in oxygen delivery from the left shift of the dissociation curve for the free (i.e., unpolymerized) hemoglobin S molecules (23,26,29,30).

Up to now, the Food and Drug Administration has approved only a single drug that inhibits sickling by this mechanism—voxelotor (previously known as GBT440) (27,31). The drug increases hemoglobin levels and reduces markers of hemolysis (27) but has not yet been shown to reduce the frequency of pain crises or reduce chronic organ damage. Voxelotor preferentially binds to the R conformation, with one molecule of the drug sitting in the pocket between the *α* chains and forming a covalent bond (Schiff’s base) to the N-terminus of one of the chains (32, 33, 34). Here, we show that slow binding and dissociation of the drug results in very different oxygen dissociation and association curves that can be readily explained with a straightforward application of the MWC model that includes drug binding. Remarkably, our set of quite complicated experimental results can be quantitatively explained by just adding drug binding and dissociation rates to the MWC model. Our analysis demonstrates the power of the MWC model and should be useful for further studies of oxygen-affinity modifiers as potential drugs to treat sickle cell disease.

## Materials and methods

### Materials and oxygen-binding measurements

Blood samples collected with an EDTA anticoagulant were obtained from a normal volunteer under National Institutes of Health protocol 08-DK-0004. The blood was diluted 100-fold into pH 7.4 phosphate-buffered saline at 300 mOsM, containing 40 mM phosphate, 115 mM sodium chloride, 5 mM dextrose, and 1 mg/mL bovine serum albumin. Oxygen dissociation and association curves at 37°C were measured with a Hemox-Analyzer (TCS, Medical Products Division, Southampton, PA). The major problem with determining oxygen dissociation and oxygen association curves with this instrument is that it assumes the saturation of hemoglobin with oxygen is 100% in room air and 0% at the lowest achieved pressure of ∼2 torr, which introduces a significant error for high-affinity binding curves that have a much higher fractional saturation than near zero at the lowest pressure. Nevertheless, as described in the Supporting materials and methods, accurate saturations could be obtained from a detailed analysis at each measured oxygen pressure of the fraction of the total optical density difference between the highest and lowest measured oxygen pressures. Although the instrument is not as accurate as the measurements made with much more sophisticated instrumentation by experts in measuring hemoglobin-oxygen binding such as Imai, Gill, Poyart, Yonetani, Rossi-Bernardi, and many others, it is sufficient for our purposes, for which big effects are observed. We should, however, point out that the p50 of ∼37 torr measured in the absence of voxelotor with the above buffer is higher than 28 torr with the buffer supplied by TCS, which is the same as the physiological p50 found for a CO_2_/bicarbonate buffer at 37°C (35). We do not yet understand the origin of this difference. Although it may result in parameters of our theoretical model that are somewhat different from parameters that would be obtained under strict physiological conditions, it does not affect any of our conclusions concerning the effect of the drug on in vitro or in vivo oxygen binding.

### Theoretical model and data fitting

Our theoretical model is based on the two-state allosteric model of Monod, Wyman, and Changeux, in which each of the two quaternary structures, R and T, can have 0–4 oxygen molecules bound, so there are 10 states in the system. The relative probability of each of these states as a function of oxygen concentration at equilibrium is given by the simple and elegant MWC partition function (*Q*):

(1) $$Q\;=\;{\left(1\;+\;K_{R}x\right)}^{4}\;+\;L{\left(1\;+\;K_{T}x\right)}^{4},$$

where *x* is the concentration of unbound oxygen in our red cells, which at equilibrium is proportional to the partial pressure of gaseous oxygen bubbling through the cell suspension according to Henry’s law (no relative of the author) in the Hemox cuvette; *K*_R_ and *K*_T_ are the equilibrium constants for oxygen binding to the R and T quaternary states; and *L* = [T_0_]/[R_0_] is the ratio of the quaternary concentrations when no oxygen is bound (*x* = 0). With one molecule of voxelotor binding to each quaternary structure (32, 33, 34) and our assumption of the drug causing no effect on the oxygen affinity of either, the partition function in the presence of drug (*Q*^*X*^) becomes

(2) $$Q^{X}\;=\;\left(1\;+\;K_{R}^{X}X\right)\;{\left(1\;+\;K_{R}x\right)}^{4}\;+\;L\;\left(1\;+\;K_{T}^{X}X\right){\left(1\;+\;K_{T}x\right)}^{4},$$

where *X* is the free drug concentration inside the red cell and $K_{R}^{X}$ and $K_{T}^{X}$ are the binding constants of the drug to the two quaternary states.

The concentrations of the now 20 states as a function of time are given by a system of 20 coupled differential equations. Rather than write out the full system of differential equations, just one of the equations is presented to illustrate the various contributions to the evolution of the population of each state. All species concentrations on the right-hand side, as well as the free oxygen and drug concentrations, are time dependent.

(3) $$\frac{d\left[R_{1}^{X}\right]\left(t\right)}{dt}\;=\;\begin{array}{ll} +\;4k_{b}^{R}\left[R_{0}^{X}\right]x & \left\{\text{oxygen}\;\text{binding}\;\text{to}\;\text{zero}\;\text{liganded}\;\text{state}\right\}\\ -\;3k_{b}^{R}\left[R_{1}^{X}\right]x & \left\{\text{oxygen}\;\text{binding}\;\text{to}\;\text{current}\;\text{state}\right\}\\ +\;2k_{d}^{R}\left[R_{2}^{X}\right] & \left\{\text{oxygen}\;\text{dissociation}\;\text{from}\;\text{doubly}\text{-}\text{liganded state}\right\}\\ -k_{d}^{R}\left[R_{1}^{X}\right] & \left\{\text{oxygen}\;\text{dissociation from current}\;\text{state}\right\}\\ +\;k_{1}\left(TX\to RX\right)\left[T_{1}^{X}\right] & \left\{\text{quaternary change}\;\text{from}\;\text{corresponding T}\;\text{state}\right\}\\ -k_{1}\left(RX\to TX\right)\left[R_{1}^{X}\right] & \left\{\text{quaternary change}\;\text{from}\;\text{current R}\;\text{state}\right\}\\ +\;k_{R}^{bX}\left[R_{1}\right]\;X & \left\{\text{binding to corresponding drug}\text{-}\text{free R state}\right\}\\ -k_{R}^{dX}\left[R_{1}^{X}\right] & \left\{\text{drug dissociation from current R state}\right\}. \end{array}$$

The numerical factors in the terms involving oxygen binding or dissociation represent statistical factors reflecting how many of the four hemes in the tetramer are available to participate in the specified transition.

The key rate coefficients that must be adjusted in fitting the model to the data are those for drug binding and dissociation in each quaternary structure. The model also contains many additional rates that are all on a second or subsecond timescale compared to the experimental timescale for dissociation and association curve measurements of many minutes. They include the known oxygen-binding and dissociation rates to T and R, assumed to be unaffected by drug binding; the known quaternary transition rate coefficients for all five ligation states without the drug bound; the quaternary rates with the drug bound; and the rates for entry of the drug into and out of the red cells. Although these rates have no effect on the fits and could have been assumed to be instantaneous (see (36)), they have been included for completeness because they could be important for future studies of oxygen-affinity-modifying drugs with faster kinetics. Also not included in the fits is the effect of drug binding to bovine serum albumin in the cell suspension buffer. This omission was done to simplify the model and make the effect of the drug more transparent. This simplification will increase the values of the binding rates compared to the fitted values and thereby decrease the dissociation constants for drug binding to hemoglobin, but it will not affect the quaternary equilibrium constants for drug-bound hemoglobin and drug dissociation rates, which are the critical parameters for explaining the increase in oxygen affinity in the presence of the drug and the time-dependent right shift of the oxygen association curve. The fitting procedure and many more details can be found in the Supporting materials and methods.

## Results

It is important to point out at the outset the very important fact that oxygen binding to hemoglobin A and hemoglobin S in the absence of polymerization was shown to be identical by Gill and co-workers (37). Consequently, all of our experiments on normal red cells apply to the unpolymerized HbS in sickle cell red cells.

### Oxygen dissociation and binding in absence of drug

**Figure 1:**
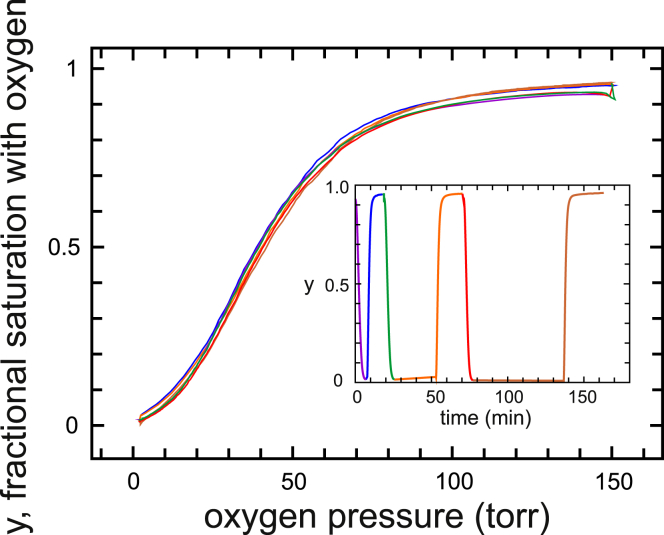
Measured oxygen dissociation and association curves in the absence of voxelotor at 37°C for normal red cells diluted 100-fold into pH 7.4 phosphate-buffered saline. The curves are fitted well (measured points not shown to reduce clutter) with the three parameters of the MWC allosteric model: *K*_R_, the affinity of oxygen for R; *K*_T_, the affinity of oxygen for T; and *L*, the concentration ratio of the two zero-liganded quaternary conformations ([T_0_]/[R_0_]) when no oxygen is present. The parameters are given in Table 1. The inset shows the saturation as a function of experimental time. If the red cells were unaffected during the time spent at 37°C in the buffer and measurements were perfect, the fractional saturation of almost zero at the end of the dissociation measurement would be exactly equal to the fractional saturation at the beginning of the association measurement, i.e., the lines connecting the beginning and end points in the inset would be perfectly horizontal (the instrument did not permit any measurements to be made during the 25 and 55 min intervals). The nearly perfectly horizontal lines during the intervals indicate that both the red cells and the instrument are reasonably stable.

Fig. 1 makes it perfectly clear what is meant by a true equilibrium oxygen-binding curve. Both the dissociation and association curves are identical, the former measured by starting at room air (∼150 torr oxygen) and decreasing the oxygen pressure and the latter measured by starting at the lowest achievable pressure (∼2 torr) and increasing the oxygen pressure. There is no significant difference between the dissociation and association curves. The inset shows a trajectory of the fractional saturation with oxygen as a function of experimental time, with intervals between the end of measuring a dissociation curve and the start of measuring an association curve of ∼1, 28, and 60 min. The continuous curves are the fits to the data obtained by varying the three parameters of the allosteric model: *K*_R_, the affinity of oxygen for R; *K*_T_, the affinity of oxygen for T; and *L*, the population ratio of the two zero-liganded quaternary conformations (T_0_/R_0_) at zero oxygen concentration. The parameters are given in Table 1.

**Table 1:** Key parameters derived from fits with model

*L*	75,000–160,000
*L*^*X*^ (with drug bound)	20
*K*_*T*_	3.5 mM^−1^ (p50 = 160 torr)
*K*_*R*_	240 mM^−1^ (p50 = 2.3 torr)
$k_{R}^{bX}$ (drug binding to R)^a^	0.02–0.03 mM^−1^ s^−1^
$k_{R}^{dX}$ (drug dissociation from R)	1–2 × 10^−4^ s^−1^
$K_{R}^{X}$	130–210 mM^−1^
$k_{T}^{bX}$ (drug binding to T)^b^	4–5 × 10^−5^ mM^−1^ s^−1^
$k_{T}^{dX}$ (drug dissociation from T)	4–8 × 10^−4^ s^−1^
$K_{T}^{X}$	0.06–0.1 mM^−1^

The complete set of parameters is given in the Supporting material. The range in the fitted parameters is obtained from multiple experimental determinations and initial conditions for searching parameter space using a *χ*^2^ criterion.

a The model assumes that the drug concentration in the red cell is driven toward equilibrium with the concentration in the buffer at all times. Therefore, at 0.200 mM drug concentration, 5.4 mM Hb tetramer concentration within the red blood cells, and 0.004 volume fraction of red blood cells in the sample, no more than 10% of the drug is depleted at any point in the reaction. Consequently, the reaction is nearly pseudo-first order with a half time for binding to R of 0.693/((0.02–0.03) × 0.2) = 120–170 s = 2–3 min. At a drug concentration of 0.012 mM, the fractional binding of R to the drug is ∼0.4 at equilibrium; a full solution of the bimolecular rate equation predicts a half time for approaching this value, from an initial value of 0, of ∼1200 s.

b Because the affinity of the drug is so low for T, the population of drug-bound T at equilibrium, even at the higher of the two concentrations 0.2 and 0.012 mM, is ∼0.01. Therefore, very little drug is consumed by binding to T hemoglobin at either concentration; a hypothetical relaxation of drug binding by T approaching the corresponding equilibrium concentration would thus be pseudo-first order, with half-times of ∼1100 and ∼11,000 min respectively.

### Oxygen dissociation and binding at near 100% modification with drug

**Figure 2:**
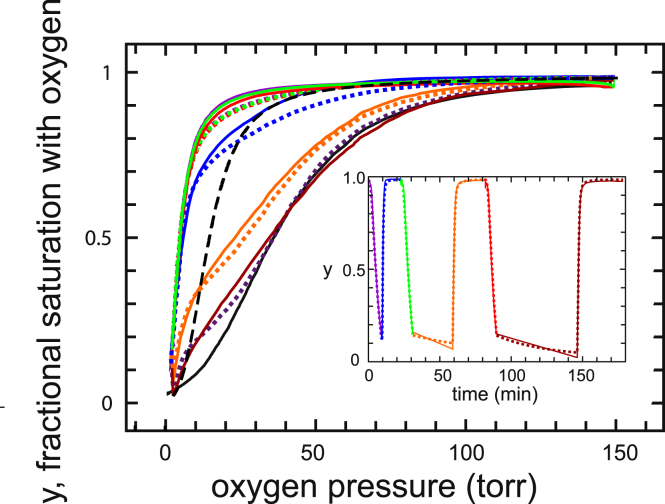
Oxygen dissociation and association curves at 37°C for normal red cells diluted 100-fold into pH 7.4 phosphate-buffered saline containing 200 *μ*M voxelotor. The points are the measured saturations, and the continuous colored curves are theoretical curves generated by the model using the allosteric parameters from the fits to the drug-free curves in Fig. 1 and varying the four rate coefficients to describe the drug binding and dissociation rates to R and T as described above and in more detail in the Supporting materials and methods. The best least-squares fit parameters are given in Table 1. The inset shows the saturation as a function of experimental time. The time at which the dissociation and association curves were measured is provided by the corresponding colors in the inset. The start of the measurement of the first oxygen dissociation curve began after incubating the red cell suspension with the drug for 1 h at 37°C. The dashed black curve is the true equilibrium curve at 200 *μ*M voxelotor, i.e., the curve that would be obtained if the drug binding and dissociation were instantaneous. The continuous black curve is the equilibrium curve in the absence of the drug (same curve as in Fig. 1).

Fig. 2 shows that the results are dramatically different with buffer containing 200 *μ*M voxelotor, a concentration high enough to produce almost 100% modification of hemoglobin with the drug. Successive measurements in which the oxygen pressure is decreased from ∼150 torr to ∼2 torr, followed by increasing the oxygen to 150 torr, resulted in three almost identical dissociation curves but three very different association curves that depend on the interval between the end of the dissociation curve measurements and the beginning of the association curve measurements. The inset for the fractional saturation with oxygen as a function of the experimental time shows that there is a large decrease in fractional saturation during the 28 and 56 min intervals at the lowest oxygen pressure. The continuous curves are the fits to the data obtained by varying the four rate parameters that describe drug binding in addition to the three allosteric parameters (Table 1). For completeness, oxygen-binding and dissociation rates were included and the quaternary rates varied, but they are subsecond for all ligation states (Table S1) and therefore have no effect on the fits to curves that are measured in minutes.

**Figure 3:**
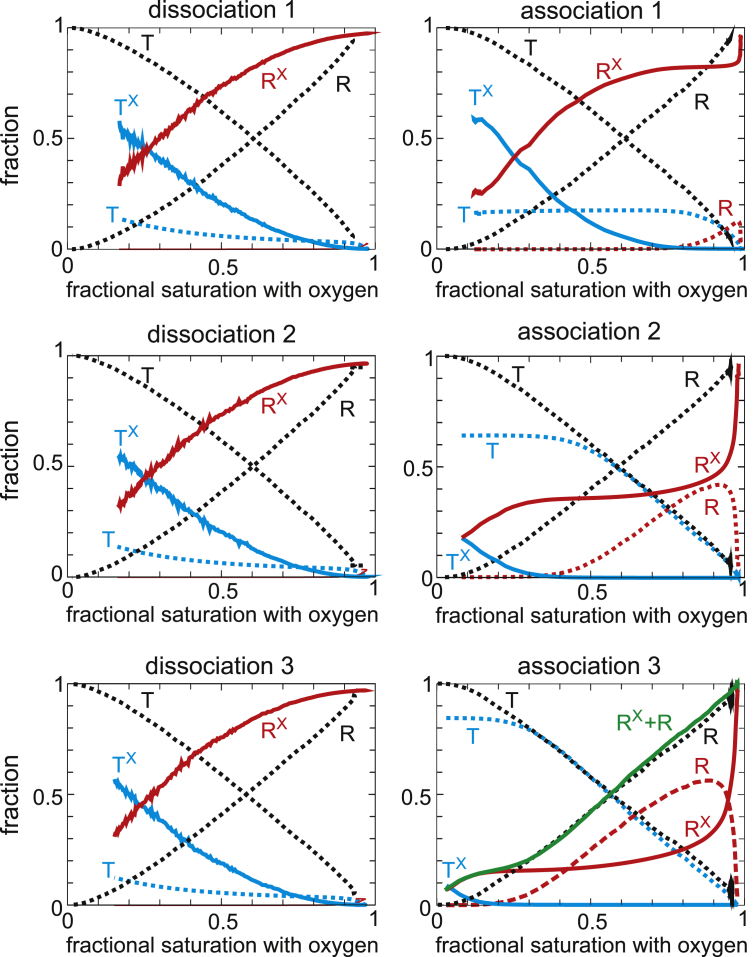
Populations of R and T conformations as a function of fractional saturation with oxygen for each of the six successive oxygen dissociation/binding curves at 200 *μ*M drug concentration obtained from the fits with the model together with the populations in the absence of drug (*black dotted lines*). The superscript X indicates that drug is bound. The green continuous curve in the “association 3” panel is the sum of the drug-free and drug-bound conformations and shows that relative populations of the total R and T populations are similar to the curves when no drug is present. The longer periods of data recording by the instrument at high saturation at the end of the association curves, as observed in the inset to Fig. 2 compared to the insets in Figs. 1 and 4, presumably result from the instrument recording until the optical density difference achieves the value before the beginning of the dissociation curve and are due to the slow association of the drug to R to form R^X^ with a half time of 2–3 min (Table 1).

The explanation of the time-dependent association curves is found in Fig. 3, which shows the populations of the R and T conformation as a function of oxygen saturation obtained from the model. As the oxygen saturation decreases in the absence of drug, there is a simple conversion of R to T. At 200 *μ*M drug (Fig. 2), R molecules with the drug bound (R^X^) are more populated than in the absence of the drug (R), which, together with the much higher (70-fold) oxygen affinity of R than T, explains why the dissociation curve in Fig. 2 is a noncooperative hyperbolic curve. The final saturation is not zero because the lowest oxygen pressure achieved with this instrument of ∼2 torr is comparable to the p50 for R of ∼2 torr. Unlike dissociation in the absence of the drug, there are two populations of T in the presence of the drug, one with drug bound (T^X^) and one from which the drug has dissociated (T). The population of T relative to T^X^ increases as the saturation decreases because of the low affinity of the drug for T (at 200 *μ*M, ∼10% of T have drug bound at equilibrium), which dissociates with a half time of ∼15 min (Table 1), in the same time regime of 8 min for measurement of the entire dissociation curve (Fig. 2, *inset*).

**Figure 4:**
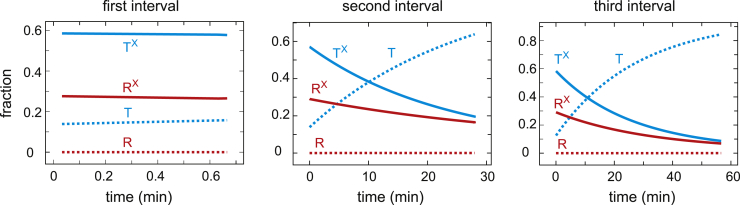
Populations of R and T conformations as a function of time in the presence of 200 *μ*M voxelotor during the three intervals between the end of the dissociation curve measurements and the beginning of the association curve measurements (see *inset* of Fig. 2). The superscript X indicates that drug is bound.

There is only a 0.7 min delay between the end of the dissociation curve and the start of the association curve, so this first association curve is only slightly right shifted from the dissociation curve because of the slightly higher population of low-affinity T (T + T^X^) conformations in association compared to dissociation. The T population does not convert to R as the saturation increases until the T^X^ population is depleted. In addition, the appearance of R^X^ from T conformations that have converted to R is delayed by the 2.5 min half time for drug binding to R. There is a large right shift of the second association curve and an even larger one for the third because of the increased T population that forms during the intervals (Fig. 4). This T population is mostly drug free (T) from the dissociation of the drug during the interval (Figs. 3 and 4). 95% of the conformational population at the beginning of the third association curve is T (Fig. 3), with the result that the curve looks very similar to the cooperative curve for red cells in the absence of drug. There is no drug binding to T during the 12 min association curve measurements because the half time for binding of ∼1100 min (Table 1) is much too slow. Consequently, T switches to drug-free R (R), which converts to drug-bound R (R^X^) with a half time of ∼2.5 min, so there is increasing conversion of R to R^X^ as the saturation increases, and by the end of the association measurements, all R has drug bound (R^X^).

### Oxygen dissociation and binding at partial modification with drug

**Figure 5:**
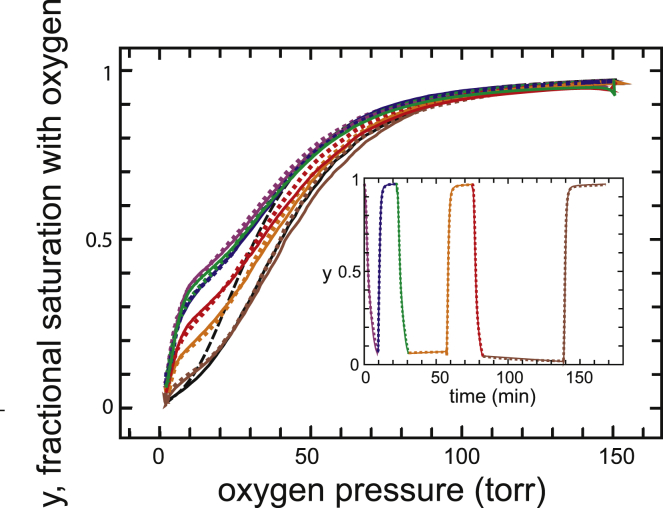
Oxygen dissociation and association curves at 37°C for normal red cells diluted 100-fold into pH 7.4 phosphate-buffered saline containing 12 *μ*M voxelotor. The points are the measured saturations, and the continuous colored curves are the fits to the data obtained by using the allosteric parameters from the fits to the drug-free curves in Fig. 1 and varying the four rate coefficients to describe the drug binding and dissociation rates to R and T. The best least-squares fit parameters, which are the same for fitting the data in Figs. 2 and 3, are given in Table 1, together with the experimental uncertainties from fits to multiple data sets at varying drug concentrations. The inset shows the saturation as a function of experimental time. The time at which the dissociation and association curves were measured is provided by the corresponding point colors in the inset. The start of the measurement of the first oxygen dissociation curve began after incubating the red cell suspension with the drug for 1 h at 37°C. The dashed black curve is the true equilibrium curve at 12 mM voxelotor, i.e., the curve that would be obtained if the drug binding and dissociation were instantaneous. The continuous black curve is the equilibrium curve in the absence of the drug (same curve as in Fig. 1).

**Figure 6:**
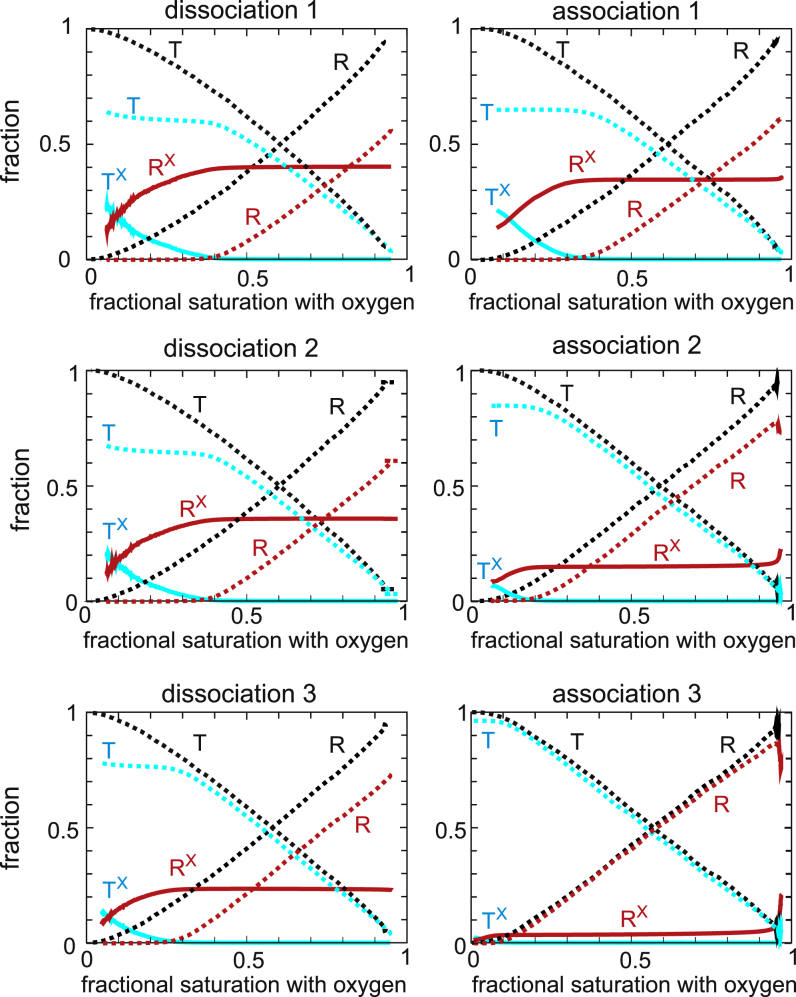
Populations of R and T conformations as a function of fractional saturation with oxygen for each of the six successive oxygen-binding curves at 12 *μ*M drug concentration obtained from the fits with the model. The superscript X indicates that drug is bound.

**Figure 7:**
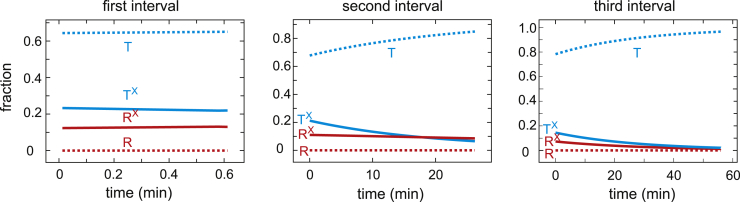
Populations of R and T conformations as a function of time in the presence of 12 *μ*M voxelotor during the three intervals between the end of the dissociation curve measurements and the beginning of the association curve measurements (see *inset* of Fig. 2). The superscript X indicates that drug is bound.

Because of the slow dissociation of the drug from hemoglobin, the measured dissociation curve at partial modification with the drug shown in Fig. 5 is biphasic (26,32,34,38,39). It consists of two distinct oxygen dissociation curves, one for the drug bound to hemoglobin and one for the drug-free hemoglobin (26,38). To be more precise, it is the fraction-weighted sum of the two curves. The explanation of the curves is the same as above for 200 *μ*M, albeit with smaller effects because of the lower fraction of molecules with drug bound. One difference is that the population of R with drug bound is less at the beginning of the second and third dissociation curves compared to the first (Fig. 6), which accounts for the apparent decrease in the high-affinity fraction most apparent in the third dissociation curve (Fig. 5). The decrease in drug-bound R occurs because of the slow binding of the drug to R, which is formed from T with no drug bound as the oxygen saturation increases in the second association curve (Fig. 7). It also occurs because there was no delay between the end of the association curve measurements and the beginning of the dissociation curve measurements.

## Discussion

A well-known general principle of thermodynamics is that the properties of a system under a specific set of conditions at equilibrium are independent of the path that is taken to attain those conditions. For the hemoglobin-oxygen system, it means that the fractional saturation of hemoglobin with oxygen at a given partial pressure of oxygen does not depend on whether that partial pressure is reached by increasing the oxygen pressure or by lowering the oxygen pressure. The identity of the dissociation and association curves shown in Fig. 1 therefore demonstrates unambiguously that they are true equilibrium curves, which has been known for almost 100 years because oxygen binding and dissociation are so much faster than the time to measure the dissociation and association curves (10,11).

In the presence of voxelotor, the dissociation and association curves are very different, and neither is an equilibrium curve. At 200 *μ*M, there is a very large difference between the dissociation and association curves, with the difference increasing as the interval increases between the end of the dissociation measurement and the beginning of the association measurement. The differences at 12 *μ*M are smaller, but the same effects are observed. We have been able to quantitatively explain this rather complicated set of results with a straightforward application of the MWC model, to which binding and dissociation rates of the drug for the R and T conformations have been added with the assumption that the drug does not alter the affinity of either R or T. The fitting process is challenging, as it not only involves varying a number of parameters to optimize the fits to the data, including four parameters to describe the quaternary transition equilibrium constants, but also determining accurate fractional saturations from the raw data reported by the instrument. Although quaternary rates were varied for completeness, they are all subsecond and have no influence on either the binding or dissociation curves. Given the simplifying assumptions of the model, the deficiencies in the instrument, and the complexity of the data, the fits in Figs. 2 and 5 can be considered impressive. They also demonstrate the enormous power of the MWC model in explaining so many different kinds of experimental results for hemoglobin without having to extend it to include tertiary conformational changes (40,41) that could be affected by drug binding.

In this case, the keys to explaining the data are the drug binding and dissociation rates for the R and T conformations obtained from the model. Although the affinity of the drug for R is so great that three successive, identical dissociation curves at 200 *μ*M drug are almost hyperbolic, the association curves are time dependent because three of the four rates for drug binding and dissociation are on the same timescale as the experiment. A detailed explanation of the dissociation and association curves in terms of the R and T conformations with and without drug bound as a function of fractional saturation with oxygen (Figs. 3, 4, 6, and 7) is given in the Results. Some of the explanation requires considerable thought because it involves the subtle interplay of the drug kinetics and the R-T equilibria.

At 12 *μ*M voxelotor concentration, ∼40% of the hemoglobin has drug bound, which is similar to the modification achieved at drug doses being used to treat patients (27,42). The dissociation curve is now biphasic, corresponding to the sum of the curves for the drug-bound and drug-free hemoglobin, and is directly relevant to oxygen delivery in vivo, which occurs on the seconds timescale. Although the higher fractional saturations produced by the drug reduce sickling, the overall left shift potentially decreases oxygen delivery in a disease in which chronic organ damage is caused by decreased oxygen delivery (23,26,30,39). We have recently shown that, except for very low oxygen pressures uncommon in vivo, the overall effect of the left shift and reduced sickling is to reduce oxygen delivery (unpublished data).

Comparison with the true equilibrium curve shows that for a drug with the same equilibrium properties but fast kinetics, oxygen delivery would be considerably greater, with a difference that increases as the final pressure in the tissues decreases. 2,3-diphosphoglycerate, for example, is a small molecule that preferentially and rapidly binds to one of the two quaternary conformations, in this case T, so that the oxygen-binding curve measured in vitro is the same as the expected in vivo binding curve (43). An important lesson from this work is that kinetics of drug binding must be considered and understood in the development of oxygen-affinity modifiers as a treatment for sickle cell disease.

## Author contributions

E.R.H. derived equations and performed calculations. J.H., K.G., B.M., and J.M.L. performed experiments. E.R.H. and W.A.E. wrote the manuscript.

## Supporting material

Document S1. Supporting materials and methods and Table S1

Document S2. Article plus supporting material

## References

[1] Mozzarelli A., Hofrichter J., Eaton W.A. (1987). Delay time of hemoglobin S polymerization prevents most cells from sickling in vivo. Science.

[2] Eaton W.A., Henry E.R., Mozzarelli A. (1999). Is cooperative oxygen binding by hemoglobin really understood?. Nat. Struct. Biol.

[3] Eaton W.A. (2020). Hemoglobin S polymerization and sickle cell disease: a retrospective on the occasion of the 70th anniversary of Pauling’s Science paper. Am. J. Hematol.

[4] Bohr C., Hasselbalch K., Krogh A. (1904). About a new biological relation of high importance that the blood carbonic acid tension exercises on its oxygen binding. Skand. Arch. Physiol.

[5] Perutz M.F. (1970). Stereochemistry of cooperative effects in haemoglobin. Nature.

[6] Szabo A., Karplus M. (1972). A mathematical model for structure-function relations in hemoglobin. J. Mol. Biol.

[7] Monod J., Wyman J., Changeux J.P. (1965). On the nature of allosteric transitions: a plausible model. J. Mol. Biol.

[8] Shibayama N., Saigo S. (1995). Fixation of the quaternary structures of human adult haemoglobin by encapsulation in transparent porous silica gels. J. Mol. Biol.

[9] Mozzarelli A., Rivetti C., Eaton W.A. (1991). Crystals of haemoglobin with the T quaternary structure bind oxygen noncooperatively with no Bohr effect. Nature.

[10] Hartridge H., Roughton F.J.W. (1923). The kinetics of haemoglobin. II. The velocity with which oxygen dissociates from its combination with haemoglobin. Proc. Roy. Soc. Lond. Ser. A.

[11] Hartridge H., Roughton F.J.W. (1925). The kinetics of haemoglobin III - The velocity with which oxygen combines with reduced haemoglobin. Proc. Roy. Soc. Lond. Ser. A.

[12] Oder E., Safo M.K., Kato G.J. (2016). New developments in anti-sickling agents: can drugs directly prevent the polymerization of sickle haemoglobin in vivo?. Br. J. Haematol.

[13] Hahn E.V., Gillespie E.B. (1927). Sickle cell anemia - report of a case greatly improved by splenectomy - experimental study of sickle cell formation. Arch. Intern. Med.

[14] Abdulmalik O., Safo M.K., Asakura T. (2005). 5-hydroxymethyl-2-furfural modifies intracellular sickle haemoglobin and inhibits sickling of red blood cells. Br. J. Haematol.

[15] Cellmer T., Ferrone F.A., Eaton W.A. (2016). Universality of supersaturation in protein-fiber formation. Nat. Struct. Mol. Biol.

[16] Eaton W.A., Hofrichter J. (1990). Sickle cell hemoglobin polymerization. Adv. Protein Chem.

[17] Henry E.R., Cellmer T., Eaton W.A. (2020). Allosteric control of hemoglobin S fiber formation by oxygen and its relation to the pathophysiology of sickle cell disease. Proc. Natl. Acad. Sci. USA.

[18] Hofrichter J. (1979). Ligand binding and the gelation of sickle cell hemoglobin. J. Mol. Biol.

[19] Hofrichter J., Ross P.D., Eaton W.A. (1974). Kinetics and mechanism of deoxyhemoglobin S gelation: a new approach to understanding sickle cell disease. Proc. Natl. Acad. Sci. USA.

[20] Yosmanovich D., Rotter M., Ferrone F.A. (2016). Calibrating sickle cell disease. J. Mol. Biol.

[21] Sunshine H.R., Hofrichter J., Eaton W.A. (1978). Requirement for therapeutic inhibition of sickle haemoglobin gelation. Nature.

[22] Abraham D.J., Perutz M.F., Phillips S.E.V. (1983). Physiological and x-ray studies of potential antisickling agents. Proc. Natl. Acad. Sci. USA.

[23] Eaton W.A., Bunn H.F. (2017). Treating sickle cell disease by targeting HbS polymerization. Blood.

[24] Beutler E. (1975). The effect of carbon monoxide on red cell life span in sickle cell disease. Blood.

[25] Abraham D.J., Mehanna A.S., Orringer E.P. (1991). Vanillin, a potential agent for the treatment of sickle cell anemia. Blood.

[26] Ferrone F.A. (2016). GBT440 increases haemoglobin oxygen affinity, reduces sickling and prolongs RBC half-life in a murine model of sickle cell disease. Br. J. Haematol.

[27] Vichinsky E., Hoppe C.C., Howard J., HOPE Trial Investigators (2019). A phase 3 randomized trial of voxelotor in sickle cell disease. N. Engl. J. Med.

[28] Sunshine H.R., Hofrichter J., Eaton W.A. (1982). Oxygen binding by sickle cell hemoglobin polymers. J. Mol. Biol.

[29] Bunn H.F., Forget B.G. (1986).

[30] Hebbel R.P., Hedlund B.E. (2018). Sickle hemoglobin oxygen affinity-shifting strategies have unequal cerebrovascular risks. Am. J. Hematol.

[31] Han J., Saraf S.L., Gordeuk V.R. (2020). Systematic review of voxelotor: a first-in-class sickle hemoglobin polymerization inhibitor for management of sickle cell disease. Pharmacotherapy.

[32] Oksenberg D., Dufu K., Archer D.R. (2016). GBT440 increases haemoglobin oxygen affinity, reduces sickling and prolongs RBC half-life in a murine model of sickle cell disease. Br. J. Haematol.

[33] Metcalf B., Chuang C., Li Z. (2017). Discovery of GBT440, an orally bioavailable R-state stabilizer of sickle cell hemoglobin. ACS Med. Chem. Lett.

[34] Strader M.B., Liang H., Alayash A.I. (2019). Interactions of an anti-sickling drug with hemoglobin in red blood cells from a patient with sickle cell anemia. Bioconjug. Chem.

[35] Horvath S.M., Malenfant A., Rossi-Bernardi L. (1977). The oxygen affinity of concentrated human hemoglobin solutions and human blood. Am. J. Hematol.

[36] Hopfield J.J., Shulman R.G., Ogawa S. (1971). An allosteric model of hemoglobin. I. Kinetics. J. Mol. Biol.

[37] Gill S.J., Benedict R.C., Wyman J. (1979). Oxygen binding to sickle cell hemoglobin. J. Mol. Biol.

[38] Li Q., Henry E.R., Eaton W.A. (2017). Kinetic assay shows that increasing red cell volume could be a treatment for sickle cell disease. Proc. Natl. Acad. Sci. USA.

[39] Shet A.S., Mendelsohn L., Thein S.L. (2019). Voxelotor treatment of a patient with sickle cell disease and very severe anemia. Am. J. Hematol.

[40] Henry E.R., Bettati S., Eaton W.A. (2002). A tertiary two-state allosteric model for hemoglobin. Biophys. Chem.

[41] Henry E.R., Mozzarelli A., Eaton W.A. (2015). Experiments on hemoglobin in single crystals and silica gels distinguish among allosteric models. Biophys. J.

[42] Patel M. (2016). Pharmakokinetics (PK) and pharmacodynamics of GBT440, a novel hemoglobin S (HbS) polymerization inhibitor for the treatment of sickle cell disease (SCD) in healthy volunteers and SCD patients. Haematologica.

[43] Marden M.C., Hazard E.S., Gibson Q.H. (1986). Testing the two-state model: anomalous effector binding to human hemoglobin. Biochemistry.

