# Supplementary material for: MWC allosteric model explains unusual hemoglobin-oxygen binding curves from sickle cell drug binding
Source: Biophys J. 2021 Apr 29;120(12):2543–51. doi: 10.1016/j.bpj.2021.04.024 (PMC8390878; doi:10.1016/j.bpj.2021.04.024)
Supplement: Document S1. Supporting materials and methods and Table S1 [file mmc1.pdf]

Biophysical Journal, Volume *120*

Supplemental information

MWC allosteric model explains unusual hemoglobin-oxygen binding curves from sickle cell drug binding

Eric R. Henry, Julia Harper, Kristen E. Glass, Belhu Metaferia, John M. Louis, and William A. Eaton

Supplementary Information for

MWC allosteric model explains unusual hemoglobin oxygen binding curves from sickle cell drug binding

by

E.R. Henry, J. Harper, K. Glass, B. Metaferia, J. M. Louis and Eaton

Uploaded March 25, 2021

I. **An MWC model for binding and dissociation of both oxygen and drug**

In order to analyze the time-dependence of the measured oxygen-binding curves in the presence of the drug, voxelotor, it is necessary to introduce a model description which incorporates binding and dissociation of drug molecules to both R and T conformations of hemoglobin. Although oxygen binding and dissociation is essentially instantaneous on the time scale of the measurements, these rates are included for completeness. For clarity, the term "ligation state" indicates the number of oxygen molecules bound to the hemoglobin tetramer in a specific model state; the terms "drug-free" and "drug-bound" indicate whether or not a state has a drug molecule bound. Moreover, throughout the model description all state populations are in units of millimolar (mM). This requires that all equilibrium binding and kinetic rate constants, whether involving O_2 or the drug, be expressed in these units.

Modeling is based on a conventional MWC formulation. In this description, the hemoglobin tetramer exists in one of ten possible states, distinguished by quaternary state (R or T) and total number of oxygen molecules bound (0-4). (These states are identified by quaternary structure with a subscript indicating ligation state. For example, the R quaternary states with a 0, 1 or 2 ligands bound are denoted by R_0, R_1, R_2, …) In the MWC model, the thermodynamic description of oxygen binding is based on the partition function (1)

$$Q = \left(1+K_R x\right)^4 + L\left(1+K_T x\right)^4$$

where $x = [O_2]_{\text{free}}$, the concentration of oxygen free in solution, K_R and K_T are the oxygen-binding affinities of the R and T quaternary states, respectively, and $L = [T_0]/[R_0]$ when $x = 0$. The relative probability of each of the ten distinguishable states is represented by a term in the expanded partition function, and the reference state—the state corresponding to the constant term 1--is R_0.

For the purpose of describing the kinetics of structural and ligand-binding events on time scales much longer than a microsecond, i.e., beyond the characteristic time scale for possible tertiary conformational changes (2), a model based on this simple enumeration of states provides the starting point. For simplicity, such a model typically includes transitions between states with the same quaternary state but different ligation states due to discrete ligand binding and dissociation events, and transitions between states with differing quaternary states but the same ligation state due to discrete quaternary structural transitions. The realization of such a model then requires in general the specification of rates for ligand binding and dissociation within each quaternary state, and rates for quaternary conformational changes in both directions (R→T and T→R) for each ligation state.

Bimolecular rates k_b^R and k_b^T, in units of $mM^{-1}s^{-1}$, are assigned for oxygen binding to hemes in the two quaternary states, as well as unimolecular rates k_d^R and k_d^T, in units of s^{-1}, for oxygen dissociation from hemes in the two quaternary states. Rates $k_i(R\rightarrow T)$ and $k_i(T\rightarrow R)$, all in units of s^{-1}, are also assigned for quaternary structural changes in each ligation state i. Taken together, these rates provide a complete description of the kinetic model, but consistency of the kinetic description with the equilibrium partition function means that these parameters are not all independent. For example, the relations between kinetic and equilibrium parameters

$$\frac{k_b^R x}{k_d^R} = K_R x, \quad \frac{k_b^T x}{k_d^T} = K_T x$$

do not require $k_d^R = k_b^R / K_R$ and $k_d^T = k_b^T / K_T$ to be variable parameters. Moreover, the quaternary conformational rates in each ligation state are related by

$$\frac{k_i(R \to T)}{k_i(T \to R)} = L\left(\frac{K_T}{K_R}\right)^i = Lc^i$$

where $c = K_T/K_R$ is often used as an alternative MWC parameter.

In order to further reduce the number of independent parameters required to specify the model, a scale parameter, *d,* which assumes a linear free energy relation, is introduced (2-4) that allows the quaternary transition rates as a function of ligation state to be written in terms of a single reference quaternary rate, for example

$$k_i(R \to T) = k_0(R \to T) / d^i$$

In practice, $k_3(R \to T)$ is used as the reference rate; the other rates are generated by scaling upward and downward by appropriate powers of d (2-4).

Drug binding to Hb is introduced with a second manifold of ten quaternary/ligation states identical to the prototype set described above but distinguished from it by having the drug molecule bound. A member of the drug-bound set of states is distinguished from its analog in the drug-free set of states by using a superscript 'X'. Since only a single drug molecule binds to the tetramer (5), albeit with different affinities in the two quaternary states, and assuming that these drug-binding properties are independent of ligation state, then an expanded partition function incorporating drug binding again has two terms corresponding to the two tetramer manifolds. However, in this case each term consists of separate factors corresponding to drug bound/unbound state and ligation state:

$$Q_{\text{full}} = \left(1 + K_R^X X\right)\left(1 + K_R x\right)^4 + L\left(1 + K_T^X X\right)\left(1 + K_T x\right)^4$$

where X is the free drug concentration, and K_R^X and K_T^X are respective binding constants of the drug to the two quaternary states. This form is justified by the equilibrium relations

$$\frac{\left[R_i^X\right]}{\left[R_i\right]} = K_R^X X, \quad \frac{\left[T_i^X\right]}{\left[T_i\right]} = K_T^X X$$

for each individual ligation state i. As a result, there are now 20 distinguishable states R_i, R_i^X, T_i, T_i^X, $i = 0,...,4$.

The usual partition-function treatment assumes that ligands are present in sufficient excess not to be depleted significantly by binding to Hb. This is true for O_2 binding. However, care must be taken for drug binding. Samples were prepared by adding a certain amount of drug to a specific volume, and this amount will not necessarily be a significant molar excess. As Hb is sequestered in the red blood cells, the available drug must partition between the external medium and the cells, in the same manner as for oxygen. However, unlike the assumed coupling of the system to a reservoir of oxygen, the finite

supply of available drug requires consideration of mass conservation during this partition. We initially discuss how to incorporate these considerations into the evaluation of the partition function, and therefore of the equilibrium binding properties of both O_2 and the drug.

Suppose some fraction V_r of the sample volume is occupied by red blood cells; the rest is buffer volume V_b, so the total volume is $V = V_r + V_b$. If the final drug concentration is X_{total}, then the total amount of drug in the sample volume is $X_{\text{total}}V$. In the extracellular buffer, the total concentration of the drug is X^b and in the red blood cells, the total concentration is the sum of the free concentration inside the cell, X^r, and the concentration of drug molecules actually bound to Hb ($[\text{Hb}]^X$). The latter may be computed at equilibrium starting from the full partition function above, adapted for intracellular conditions and written explicitly as a function of the free oxygen concentration x:

$$Q_{\text{intra}}^{\text{full}}(x) = \left(1+K_R^X X^r\right)\left(1+K_R x\right)^4 + L\left(1+K_T^X X^r\right)\left(1+K_T x\right)^4$$

The terms from this corresponding to tetrameric states with drug bound are

$$\begin{aligned} Q_{\text{intra}}^{X}(x) &= K_R^X X^r \left((1+K_R x\right)^4 + LK_T^X X^r \left((1+K_T x\right)^4 \\ &= X^r\left[K_R^X \left((1+K_R x\right)^4 + LK_T^X \left((1+K_T x\right)^4\right] \end{aligned}$$

The fraction of Hb with drug bound is then given by

$$f_{\text{bound}}(x) = \frac{[\text{Hb}]^X}{[\text{Hb}]_{\text{total}}} = \frac{Q_{\text{intra}}^{X}(x)}{Q_{\text{intra}}^{\text{full}}(x)}$$

Note that this fraction is itself a nonlinear function of X^r, and its evaluation requires specifying values for the partition-function parameters L, K_R and K_T for oxygen binding, and K_R^X and K_T^X for drug binding.

Mass conservation requires

$$X_{\text{total}}V = X^b V_b + \left(X^r + [\text{Hb}]^X\right)V_r$$

If the cells have a fractional volume occupancy of ρ (~0.4 for undiluted blood), so that $V_r = \rho V$ and $V_b = (1\text{-}\rho)V$,

$$X_{\text{total}} = X^b(1-\rho) + \left(X^r + [\text{Hb}]_{\text{total}} f_{\text{bound}}(x)\right)\rho$$

$[\text{Hb}]_{\text{total}}$, X_{total} and ρ are experimental parameters fixed at the beginning of the analysis. In the absence of any transport mechanism which might maintain a gradient of free drug concentrations between the intracellular and extracellular regions, $X^b = X^r$ at equilibrium. (This assumption is relaxed when considering the kinetic formulation below.) Then the above is a nonlinear equation in X^r (because of the presence of $f_{\text{bound}}(x)$), which may be solved for the intracellular free drug concentration at equilibrium. This concentration is then used to evaluate the Hb partition function and equilibrium quantities derived from it, including fractional saturations of Hb with oxygen and fractions of Hb with drug bound.

The kinetic description of the expanded model has two components: the interconversion of states within the individual drug-free and drug-bound sets of states, and transitions linking analogous states

in the two sets due to binding and dissociation of the drug. The former component is straightforward, with the connectivity of the states (and the notation for the rates) in the drug-free set identical to that for the prototype model; the connectivity of the states in the drug-bound set is exactly equivalent, except that in general the transition rates in the latter set are both notationally and numerically distinct from the corresponding rates in the drug-free set. Equilibrium and kinetic parameters applicable to the drug-bound set of states are denoted by an additional subscript or suffix '*X*' applied to the corresponding parameters in the drug-free set.

In general terms, this leads to distinct binding affinities of oxygen to hemes in drug-bound tetramers, K_{RX} and K_{TX}, distinct kinetic parameters for oxygen binding and dissociation, k_{bX}^{R} , k_{bX}^{T} , k_{dX}^{R} , and k_{dX}^{T} , and distinct quaternary interconversion rates $k_i(RX \rightarrow TX)$ and $k_i(TX \rightarrow RX)$. As with the drug-free set, a number of simplifications are possible. The most important simplification reflects a fundamental assumption of the model that binding and dissociation of the drug affects the oxygen-binding properties of the tetramer only by altering the equilibrium between quaternary states, and has no direct effect on equilibria and rates of oxygen binding by hemes in tetramers in either quaternary state. As a consequence, the set of independent model parameters is significantly reduced by setting $K_R^X = K_R$, $K_T^X = K_T$, $k_{bX}^R = k_b^R$, and so forth.

Because interconversion between quaternary structures is affected by drug binding to the tetramer, distinct such rates $k_i(RX \rightarrow TX)$ and $k_i(TX \rightarrow RX)$ are maintained for the drug-bound set. The differential stabilization of quaternary states caused by stronger drug binding to the R state is reflected in a modified relation between quaternary rates for each ligation state:

$$\frac{k_i(RX \rightarrow TX)}{k_i(TX \rightarrow RX)} = L\left(\frac{K_T}{K_R}\right)^i \frac{K_T^X}{K_R^X} = Lc^i c_X = L_i^X$$

where the parameter c_X is introduced to represent the relative drug-binding affinities of the T and R quaternary states and L_i^X is the ratio of T to R concentrations with *i* oxygen molecules bound and with the drug bound (i.e., *TX*/*RX*)

In a manner similar to that employed for the drug-free set of states, a scale parameter, d_X, is introduced, which allows quaternary transition rates within the drug-bound set of states to be written as a function of ligation state in terms of a single reference quaternary rate, for example

$$k_i(RX \rightarrow TX) = k_0(RX \rightarrow TX) / d_X^i$$

As for the drug-free set of states, the rate for triply-liganded states $k_3(RX \rightarrow TX)$ is used as the reference rate, with the other rates generated by scaling upward and downward by the appropriate power of d_X.

The second set of transitions involves binding or dissociation of the drug to the Hb tetramer and therefore connects states in the drug-free set with states in the drug-bound set. As with oxygen binding, it is assumed that these transitions involve discrete drug binding or dissociation events without a change in quaternary or ligation state. It is further assumed that these binding properties depend only on the quaternary state, and not on the ligation state, of the molecule; this assumption is already implicit in the simple form of the partition function above, specifically the use of drug-binding equilibrium constants K_R^X and K_T^X . The kinetic description of drug binding/dissociation events then requires bimolecular drug association rate constants k_R^{bX} and k_T^{bX} (in units of $mM^{-1}s^{-1}$) and unimolecular drug dissociation rate constants k_R^{dX} and k_T^{dX} (in units of s^{-1}). The usual relations

between the kinetic and equilibrium constants then apply, specifically $k_R^{dX} = k_R^{bX} / K_R^X$ and $k_T^{dX} = k_T^{bX} / K_T^X$.

The basic kinetic formulation of the model consists of twenty dynamical variables, corresponding to populations of the 20 tetrameric states of the model, indexed by quaternary and ligation states and by whether or not a drug molecule is bound. All of the kinetic processes discussed so far take place inside the red blood cell. Many of these processes depend on the free concentration of drug inside the cell, X^r , which is itself a variable which depends on the instantaneous amount of drug actually bound to Hb. The simplest approach to managing the bookkeeping involving this parameter is to treat it as an additional dynamical variable operative inside the red blood cell, which changes with time due to binding and dissociation of the drug to intracellular Hb as well as possible interchange with the available drug in the extracellular buffer medium. It is straightforward to quantitatively account for the former processes using bimolecular and unimolecular rate terms, but the latter require some mass-conservation considerations akin to those applied to the partition function above.

The assumption of $X^b = X^r$ at equilibrium noted above facilitates a direct evaluation of this parameter from mass conservation and subsequent evaluation of the overall partition function. However, it is not necessary, and in fact may be an oversimplification, to assume that this equality is true at all times as the dynamical system evolves. A straightforward approach to including possible exchange of free drug between the red blood cell and the external buffer medium, which insures the eventual approach to equality of the two quantities as the system approaches equilibrium, adds a simple relaxation term to the expression for the evolution of X^r , for example

$$\frac{dX^r(t)}{dt} = \left\{\text{terms describing drug interactions with Hb}\right\} + \kappa\left(X^b(t) - X^r(t)\right)$$

for some relaxation rate κ (units s^{-1}), which may be treated as either a control parameter or an adjustable fitting parameter; this relaxation rate is a measure of the tightness of the coupling between the instantaneous free drug concentrations inside and outside the cell. Evaluation of this expression requires being able to determine the instantaneous value of X^b as a function of all the time-dependent dynamic variables. Guidance for this may be found in the mass-conservation discussion above. Note that the time-dependent contribution of drug-bound Hb to the total intracellular concentration of the drug, denoted by $[\text{Hb}]_{\text{drug bound}}(t)$, may be evaluated by summing the current populations of all the model states with drug bound. Then the mass conservation of drug becomes

$$X_{\text{total}} = X^b(t)(1-\rho) + \left(X^r(t) + [Hb]^X(t)\right)\rho$$

which may be solved for X^b :

$$X^b(t) = \frac{X_{\text{total}} - \left(X^r(t) + [\text{Hb}]^X(t)\right)}{1-\rho}$$

The evolution of the 21 dynamical variables, the populations of the 20 model states plus the intracellular free drug concentration, is described by a system of 21 simultaneous ordinary differential equations in these 21 unknowns. Equation (3) in the main text is an example of one of these differential equations. This system is parametrized by the various rate constants described above and itemized in Table S1. An important driver of the interconversion of states involving oxygen binding is x ($[O_2]_{\text{free}}$), the concentration of free oxygen; in the treatment of a conventional kinetic experiment, this quantity is either fixed, reflecting the presence of oxygen in large molar excess, or is computed

dynamically based on an assumed total concentration combined with the instantaneous populations of all model states with oxygen bound. In the experiments being treated here, which consist of sequences of deoxygenation and reoxygenation runs, the instantaneous free oxygen pressure is in fact determined by the measuring instrument and may be viewed as a time-dependent control parameter $x(t)$ wherever it appears in the differential-equation model. (Unlike the case of drug binding, the instrument-controlled free oxygen concentration equilibrates effectively instantaneously between the extracellular and intracellular media; dispensing with this assumption would require straightforward modification of the treatment of the intracellular oxygen concentration similar to that implemented for the free drug concentration.)

The analysis of a specific set of measurements using this kinetic framework requires specification of the experimental parameters $[Hb]_{total}$, X_{total} and ρ. Values of the various rate parameters are also required; only the free parameters listed in Table S1 are varied in the fits; the remaining derived parameters are automatically computed as described in the Table. In addition to these parameters, solution of the system of equations over the experimental time range requires initial ($t = 0$) populations of all states, which are computed from the equilibrium partition function evaluated at the starting oxygen concentration x ($[O_2]_{free}$) for the experiment.

Table S1. Parameters of the kinetic model. Free parameters represent the minimal set of independent parameters required to fully define the system; numerical free-parameter values marked with an asterisk are fixed at the listed values, estimated from previous studies. Derived parameters are computable from the free parameters by means of thermodynamic and scaling relations. Values given as ranges reflect the values produced by fits to distinct sets of measurements.

free parameters		
L	75,000-160,000	MWC parameter = $[T_0]/[R_0]$ at zero oxygen saturation
c	0.014	MWC parameter = K_T/K_R
K_R	240-300 mM^{-1}	O_2 binding affinity to drug-free or drug-bound R
k_b^R	33,000 $mM^{-1}s^{-1}$ *	bimolecular rate of O_2 binding to drug-free or drug-bound R
k_b^T	4,400 $mM^{-1}s^{-1}$ *	bimolecular rate of O_2 binding to drug-free or drug-bound T
$k_3(R\rightarrow T)$	500 s^{-1} *	quaternary transition rate from triply-liganded drug-free R
d	5 *	scaling between R→T rates of different ligation states in drug-free R
K_R^X	130-210 mM^{-1}	binding affinity of drug to R quaternary state
c_X	3-8×10^{-4}	relative drug-binding affinities to T and R = K_T^X / K_R^X
k_R^{bX}	0.02-0.03 $mM^{-1}s^{-1}$	bimolecular rate of drug binding to R
k_T^{bX}	4-5×10^{-5} $mM^{-1}s^{-1}$	bimolecular rate of drug binding to T
$k_3(RX\rightarrow TX)$	0.5-3 s^{-1}	quaternary transition rate from triply-liganded drug-bound R
d_X	10-50	scaling between R→T rates of different ligation states in drug-bound R
κ	0.05-0.5 s^{-1}	relaxation rate for equilibration of intra- and extracellular drug concentrations
Derived parameters		
K_T	3-5 $mM^{-1}s^{-1}$	O_2 binding affinity to drug-free or drug-bound T = cK_R
k_d^R	100-140 s^{-1}	O_2 dissociation rate from drug-free or drug-bound R = k_b^R $/K_R$
k_d^T	1000-1300 s^{-1}	O_2 dissociation rate from drug-free or drug-bound T = k_b^T $/K_T$
$k_i(R\rightarrow T)$, i=0,1,2		quaternary transition rate from i-liganded drug-free R = d^{3-i} $k_3(R\rightarrow T)$
$k_4(R\rightarrow T)$	100 s^{-1}	quaternary transition rate from 4-liganded drug-free R = $k_3(R\rightarrow T)/d$
$k_i(T\rightarrow R)$, i=0-4		quaternary transition rate from i-liganded drug-free T = $k_i(R\rightarrow T)/(Lc^i)$
K_T^X	0.06-0.1 mM^{-1}	binding affinity of drug to T quaternary state = c_X K_R^X
k_R^{dX}	1-2×10^{-4} s^{-1}	rate of drug dissociation from R = k_R^{bX} / K_R^X
k_T^{dX}	4-8×10^{-4} s^{-1}	rate of drug dissociation from T = k_T^{bX} / K_T^X
$k_i(RX\rightarrow TX)$, i=0,1,2		quaternary transition rate from i-liganded drug-bound R = d_X^{3-i} $k_3(RX\rightarrow TX)$
$k_4(RX\rightarrow TX)$	0.05-0.07 s^{-1}	quaternary transition rate from 4-liganded drug-bound R = $k_3(RX\rightarrow TX)/d_X$
$k_i(TX\rightarrow RX)$, i=0-4		quaternary transition rate from i-liganded drug-bound T = $k_i(RX\rightarrow TX)/(L\ c_X\ c^i)$

II. Application of the model to the analysis of the experimental curves

A single oxygen dissociation or association curve measured with the Hemox instrument varies the oxygen pressure through a set of values spanning the range of ~2 torr to ~150 torr; in a deoxygenation run, the pressure begins at the upper value and is decreased in a stepwise fashion to the lower value, whereas for measurement of an oxygen association curve the pressures slew in the opposite direction. A single measurement consists of a ΔOD value (the difference between optical densities of the sample measured at two distinct wavelengths) coupled with a current value of the pressure determined from an oxygen electrode; the full data set from a single run consists of a large sequence of such (pressure, ΔOD) pairs. To determine kinetic effects requires being able to associate an experimental time with each such pair. The measurements are recorded at regular time intervals; however, the instrument does not generate a timestamp for each measurement, so it was necessary to deduce a time interval between successive measurements by manually measuring the (clock) time required to perform a known number of measurements. The time increment deduced in this way varies between ~1.02 and ~1.04 seconds, this variability introducing an overall small "jitter" into the sets of times computed for each run.

A single full experiment on a sample prepared at a specific overall drug concentration (X_{total}) consisted of a series (generally three) of cycles of a deoxygenation run followed by a reoxygenation run, over a period of hours. In order to probe kinetic effects, variable waiting periods were introduced between the deoxygenation and reoxygenation runs in each cycle; during these periods, the oxygen pressure was held at the lowest value reached by the instrument (~2 torr) at the end of the deoxygenation run until commencement of the reoxygenation run. The set of (pressure, ΔOD) measurements for each run was recorded in a separate file, along with a single timestamp indicating the start time for the run. This set of timestamps, along with the estimated time increment per measurement deduced for each run, were used to produce a single global set of laboratory times corresponding to the full set of measurements from all the runs in the experiments.

This process of splicing together the measurements from the full series of runs yielded a set of thousands of (time, pressure, ΔOD) triplets, all on a consistent laboratory time scale. Because of the variable wait periods inserted at points in the experiment, this raw set of data exhibited gaps in the set of times, and therefore in the corresponding (pressure, ΔOD) measurements. In order to present a more uniform set of times to the subsequent analysis, a set of interpolated (pressure, ΔOD) pairs was created on a grid of times spanning each wait period, by linearly interpolating between final values from the run preceding the wait period and initial values from the subsequent run.

In order to subject this complete set of measurements to subsequent analysis, it was helpful to be able to sample all the results from all runs in an experiment on a uniform grid of times. This was accomplished by least-squares fitting each variable (pressure and ΔOD) to a piecewise-polynomial representation in time, which could then be evaluated at any desired times. This approach had the added advantage of suppressing the noise present in the raw pressure and ΔOD values output by the instrument.

A conventional measurement in a clinical laboratory of Hb oxygen-binding curves using this instrument generally assumes that the observed set of ΔOD values, measured over the range of pressures provided by the instrument, corresponds to very nearly the complete range of possible fractional saturations (i.e., from 0 to 1). However, it was immediately apparent that the ranges of ΔOD values exhibited during different deoxygenation/reoxygenation cycles varied to the extent that naively mapping the ranges seen in different runs to the same idealized range of saturations could not be valid, particularly for oxygen dissociation curves having a high affinity similar to the value for the R

conformation. Because a meaningful analysis of these experiments hinges on having reasonably quantitative estimates of saturations available, a hybrid model-aided approach was required.

A simple model-aided approach was helpful to improve the accuracy of saturations over those provided by the naive assumption that the observed range of ΔOD values corresponds to the full saturation range from 0 to 1. The attraction of such an approach arises partly from the fact that any reasonable assumption of a functional form for a binding curve (i.e., saturation-vs-pressure), whether it be a simple empirical expression (e.g., a Hill function), or an expression based on a thermodynamic model (e.g., a MWC binding function), automatically excludes saturation values of exactly 0 or 1. In applying such an analysis, we rely on the usual assumption that the relationship between ΔOD values and actual saturations is linear, so that the shape of the ΔOD-vs-pressure curve is the same as that of the saturation-vs-pressure curve to within a linear scaling and a uniform shift. With this assumption, the model-based analysis proceeds by simultaneously varying parameters associated with the assumed functional form for the binding curve, possibly within established ranges, and linear scaling and shift parameters, so that the scaled-and-shifted computed binding curve optimally reproduces the observed ΔOD-vs-pressure curve. Assuming that the model used is adequately descriptive of the binding curves in question, such an optimization procedure offers the simultaneous benefits of a parametrization of the model along with improved estimates of the saturations themselves.

This simple approach, which requires a uniform model description of the binding curve, failed in the present case, because the overall shape of the ΔOD-vs-pressure curve evolves dramatically between successive runs; this means that no single saturation-vs-pressure curve can be applicable to all runs. This observation is *prima facie* evidence of the role of kinetic effects in determining the evolving shape of the measured curves. We therefore adopted an approach in which a full kinetic-model-based description of the evolving relationship between pressure and saturation replaces a simple equilibrium model relating the two quantities.

Applying this approach to a single data set—i.e., created from data measured on a sample with a single known value of X_{total}—requires varying a subset of the free parameters in the kinetic model, while assigning reasonable fixed values to the remaining free parameters, and simultaneously varying linear fit parameters (scale and uniform shift), such that the overall saturation of Hb by oxygen predicted by the model, evaluated over the complete set of experimental times assembled for the data set, optimally matches the full evolution of ΔOD-vs-time over the set of runs in the experiment, when scaled and shifted by the linear fit parameters. For a specific set of model parameters, the required saturations as a function of time are easily computed from the time-dependent populations of the model states generated by the solution of the system of differential equations, weighted by the degree of ligation (number of oxygens bound) of each state. Moreover, prior to any solution of the kinetic model, a fully consistent set of parameters is maintained by evaluating all the derived parameters from the current set of free parameters.

The analysis is conducted using a standard Marquardt-Levenberg nonlinear least-squares fitting procedure, in which the adjustable parameters are varied in a systematic fashion in order to minimize the deviations between the experimental time-dependent ΔOD values and a set of corresponding simulated estimates. The latter are computed at each step by solving the full system of kinetic equations over the set of experimental times using the current set of model parameters, deriving a set of time-dependent oxygen saturations from the resulting populations of the various model states, and scaling and shifting the result using the current linear scaling and shift parameters.

It was also possible to apply this model-aided analysis in a simultaneous fashion to multiple data sets measured using the same blood sample but different total drug concentrations X_{total}. In this case, at each step of the overall fitting procedure the simulated estimates of the experimental ΔOD values were computed independently for each data set by solving the system of kinetic equations over the

set of experimental times from that data set, using the same current set of model parameters while adjusting only the value of X_{total} as appropriate for each data set; the resulting set of time-dependent oxygen saturations derived from the solution for each data set was then scaled and shifted using adjustable linear parameters maintained separately for each data set. The output of the fitting procedure was thus a single optimal set of adjustable model parameters consistently applied to all data sets, along with individual optimal linear scaling and shift parameters for each data set.

References

1. Monod J, Wyman J, & Changeux JP (1965) On the nature of allosteric transitions: a plausible model. *J. Mol. Biol.* 12(1):88-118.
2. Henry ER, Bettati S, Hofrichter J, & Eaton WA (2002) A tertiary two-state allosteric model for hemoglobin. *Biophys. Chem.* 98(1-2):149-164.
3. Sawicki CA & Gibson QH (1976) Quaternary conformational changes in human hemoglobin studied by laser photolysis of carboxyhemoglobin. *J. Biol. Chem.* 251(6):1533-1542.
4. Henry ER, Jones CM, Hofrichter J, & Eaton WA (1997) Can a two-state MWC allosteric model explain hemoglobin kinetics? *Biochemistry* 36(21):6511-6528.
5. Strader MB*, et al.* (2019) Interactions of an anti-sickling drug with hemoglobin in red blood cells from a patient with sickle cell anemia. *Bioconj. Chem.* 30(3):568-571.
